# Supplementary figures and images for: REST Targets JAK–STAT and HIF-1 Signaling Pathways in Human Down Syndrome Brain and Neural Cells
Source: Int J Mol Sci. 2023 Jun 10;24(12):9980. doi: 10.3390/ijms24129980 (PMC10298578; doi:10.3390/ijms24129980)

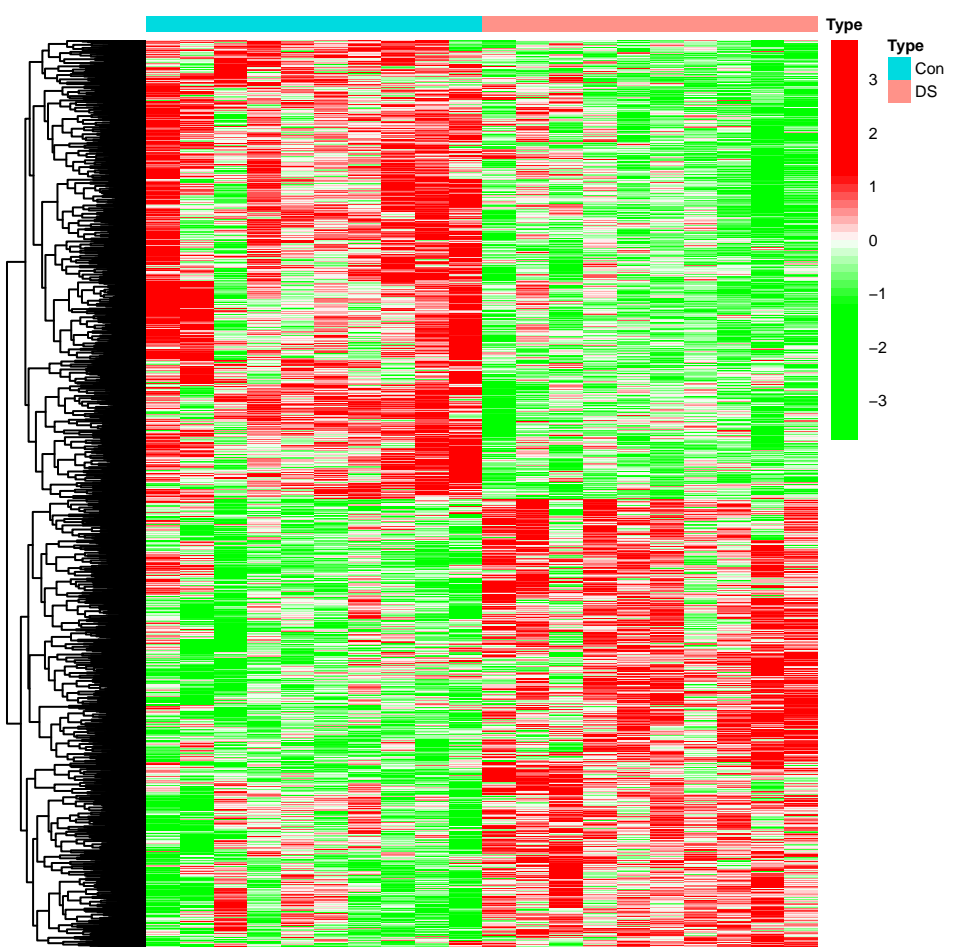

Supplement: Supplementary file 1 [file ijms-24-09980-s001.zip › Figure S1_a_CBC.pdf]

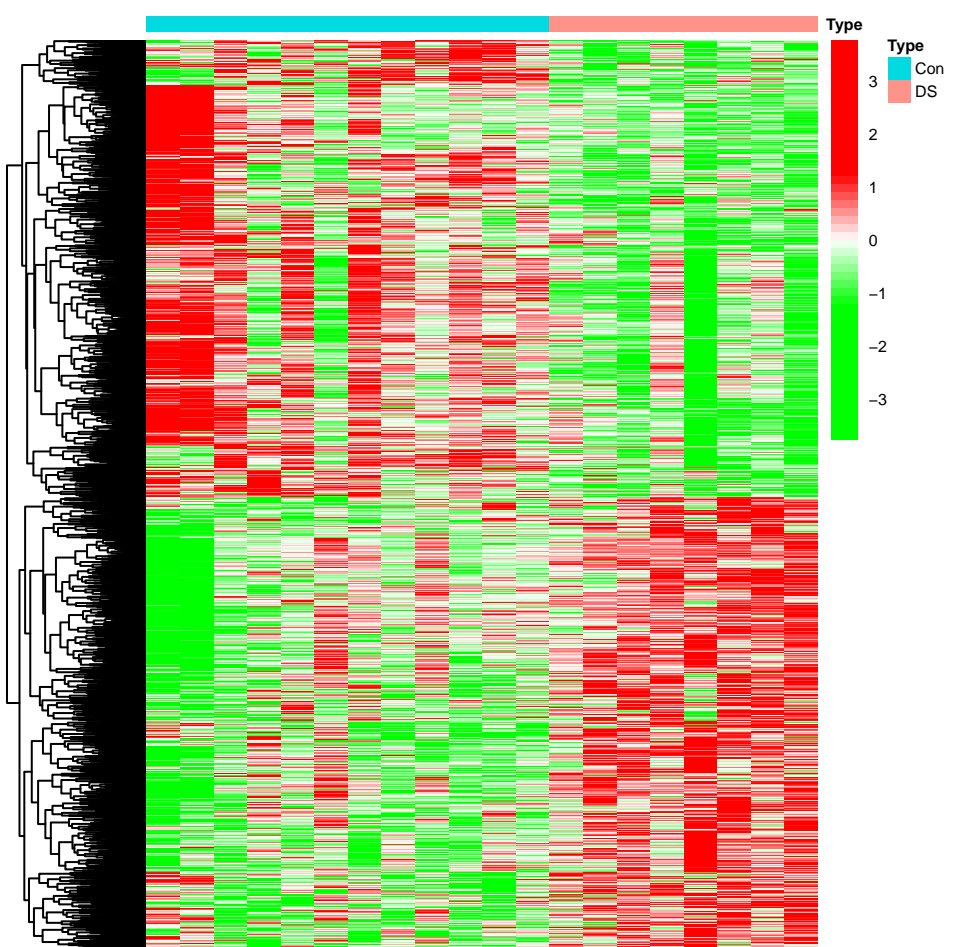

Supplement: Supplementary file 1 [file ijms-24-09980-s001.zip › Figure S1_b_DFC.pdf]

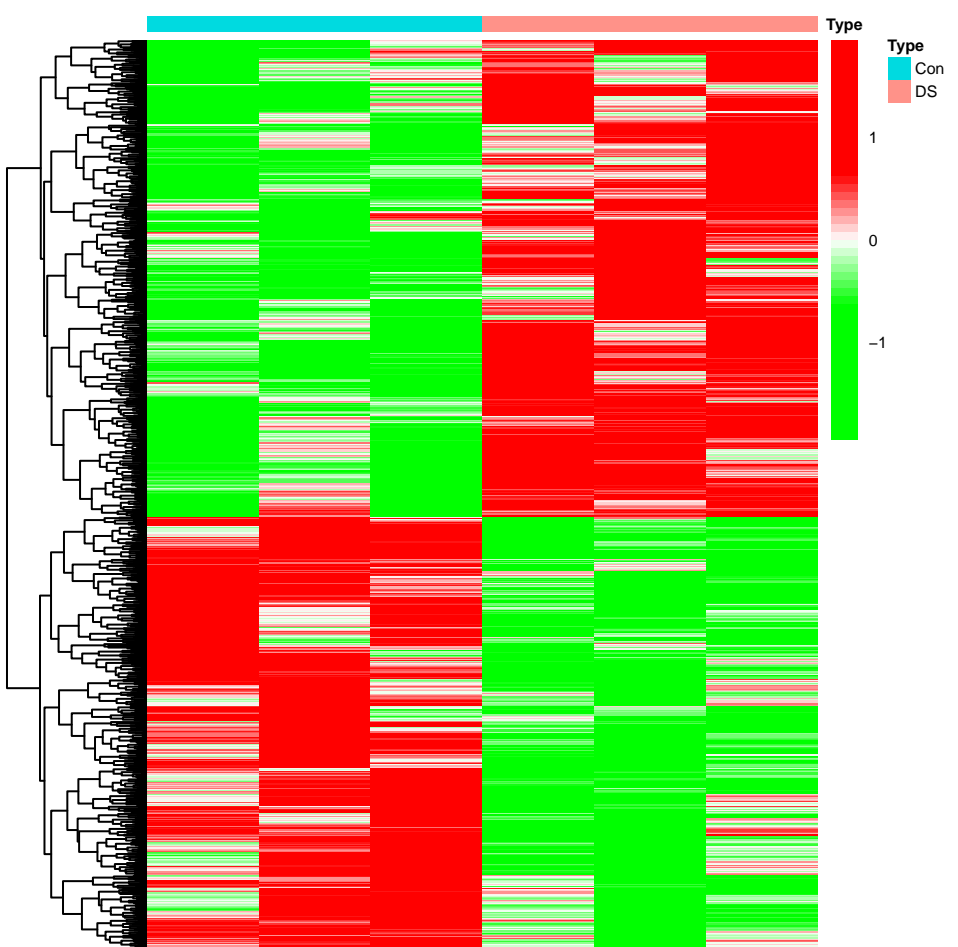

Supplement: Supplementary file 1 [file ijms-24-09980-s001.zip › Figure S1_c_HIP.pdf]

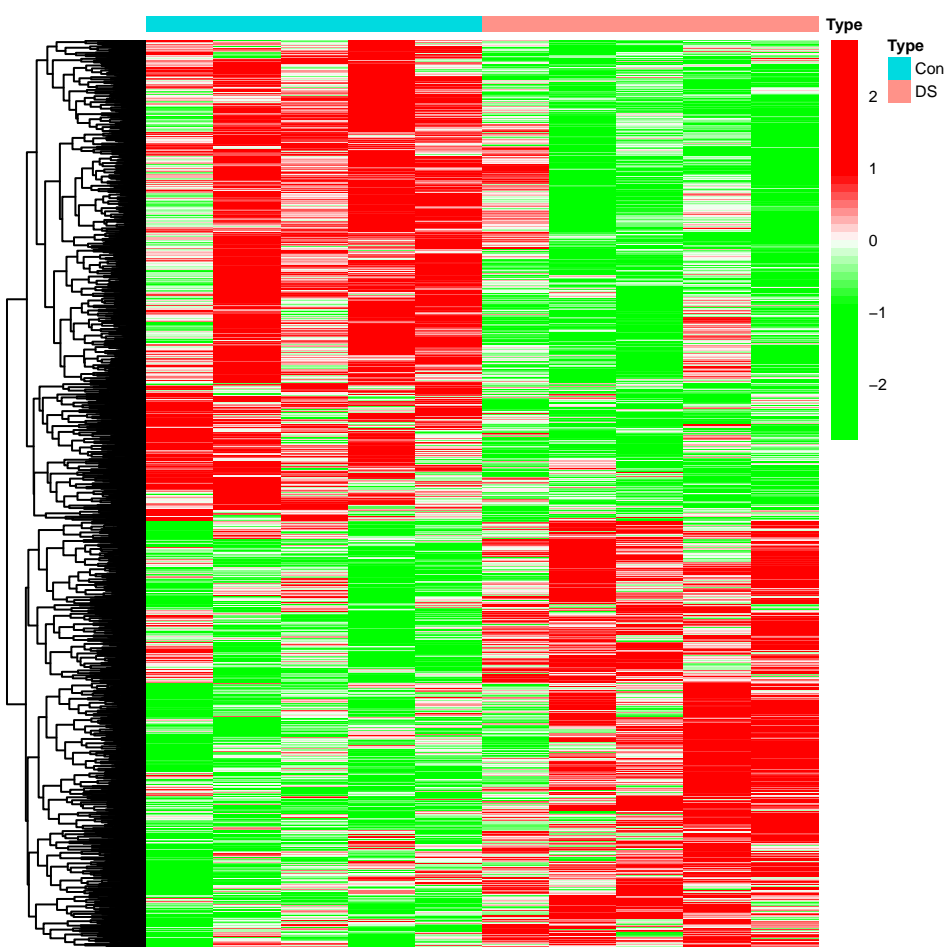

Supplement: Supplementary file 1 [file ijms-24-09980-s001.zip › Figure S1_d_ITC.pdf]

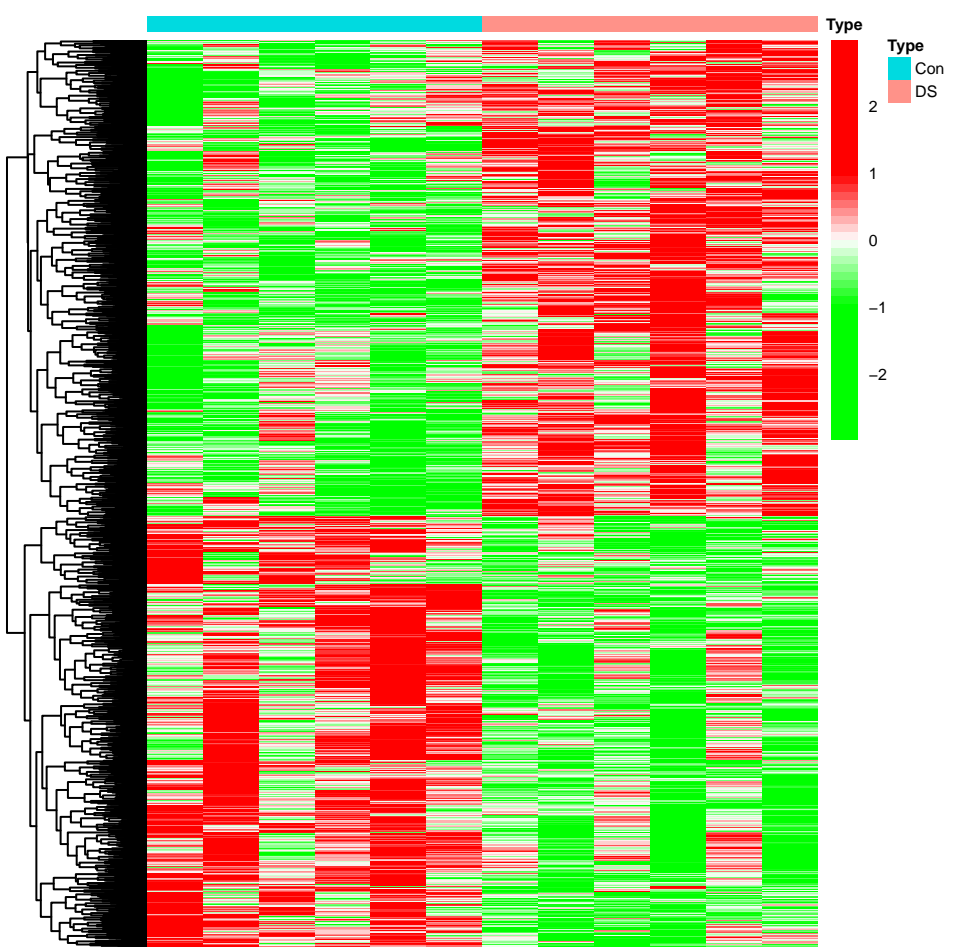

Supplement: Supplementary file 1 [file ijms-24-09980-s001.zip › Figure S1_e_OFC.pdf]

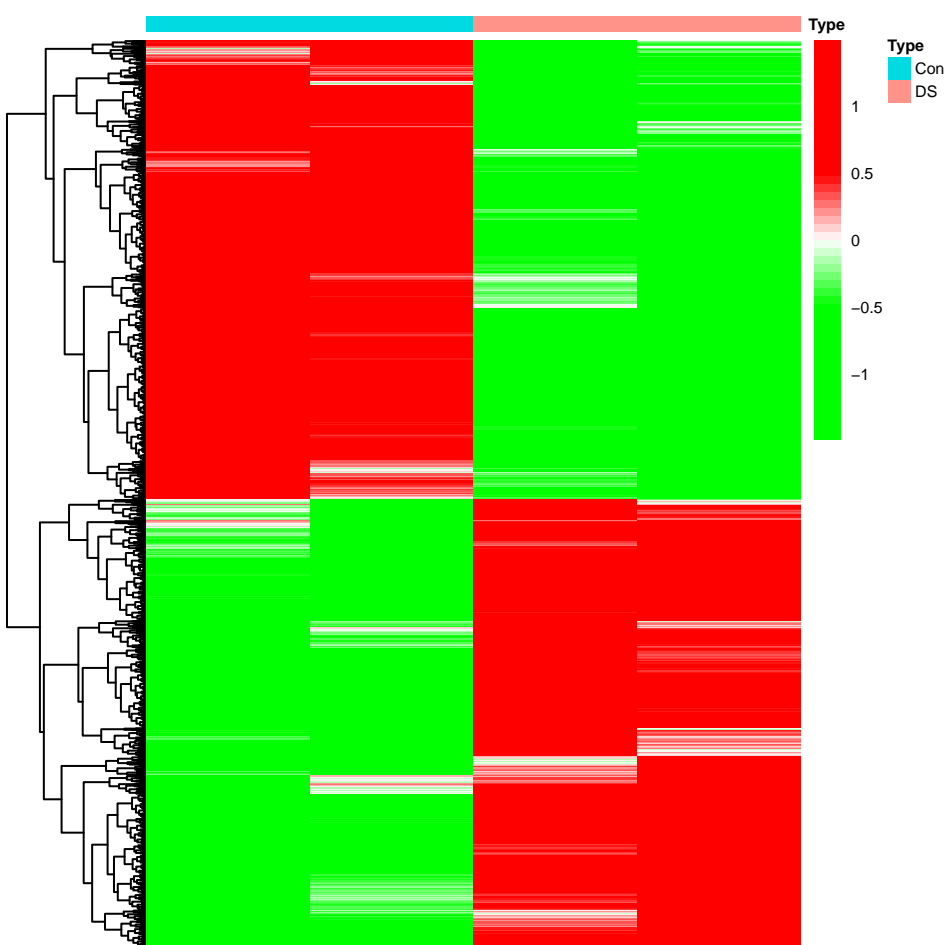

Supplement: Supplementary file 1 [file ijms-24-09980-s001.zip › Figure S1_f_S1C.pdf]

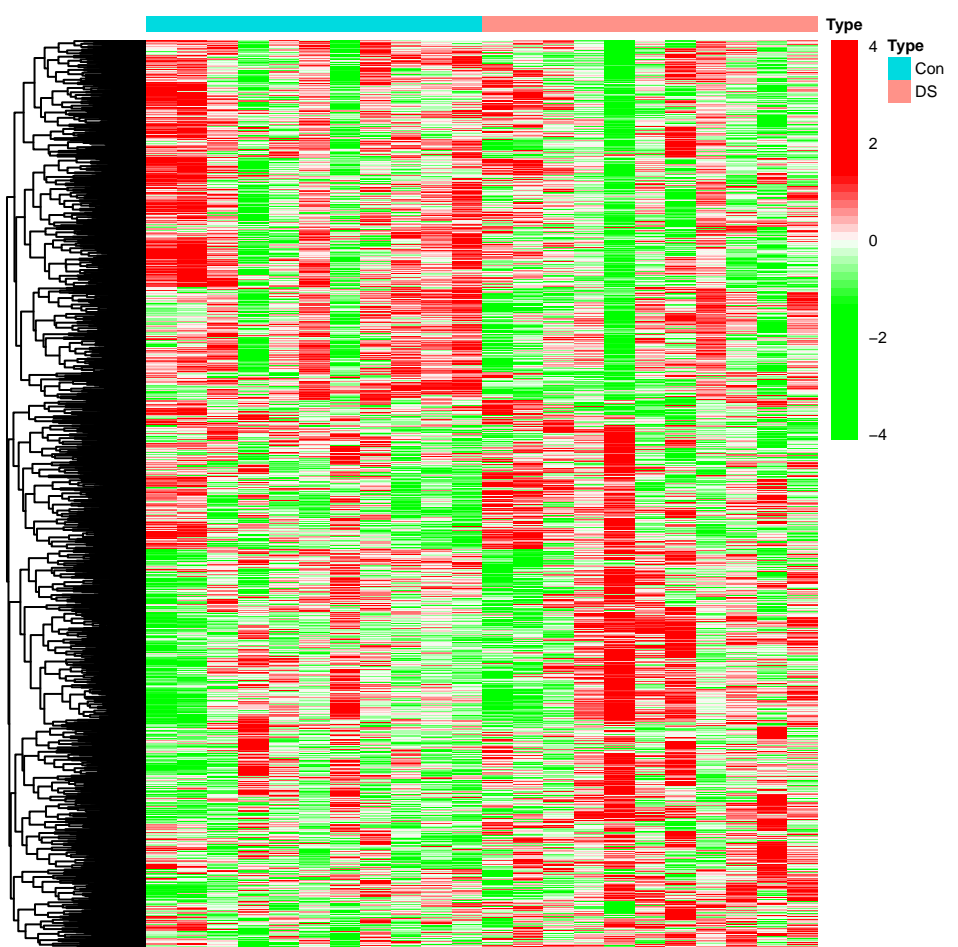

Supplement: Supplementary file 1 [file ijms-24-09980-s001.zip › Figure S1_g_V1C.pdf]

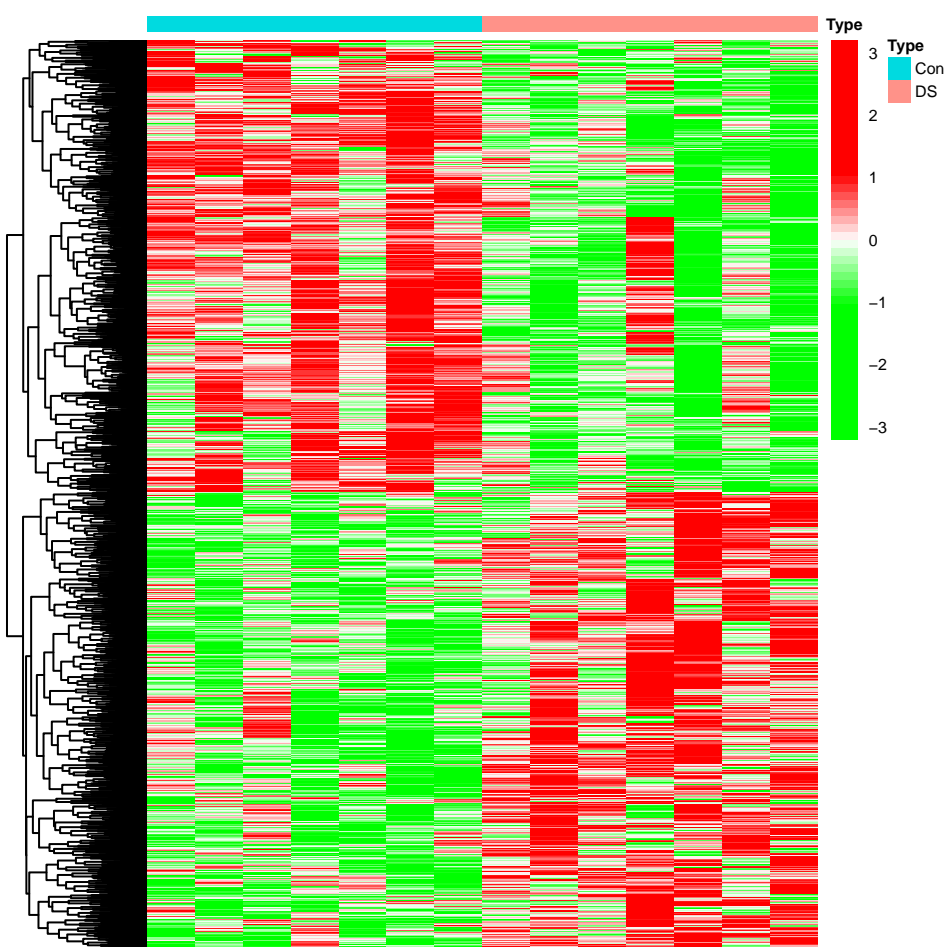

Supplement: Supplementary file 1 [file ijms-24-09980-s001.zip › Figure S1_h_VFC.pdf]

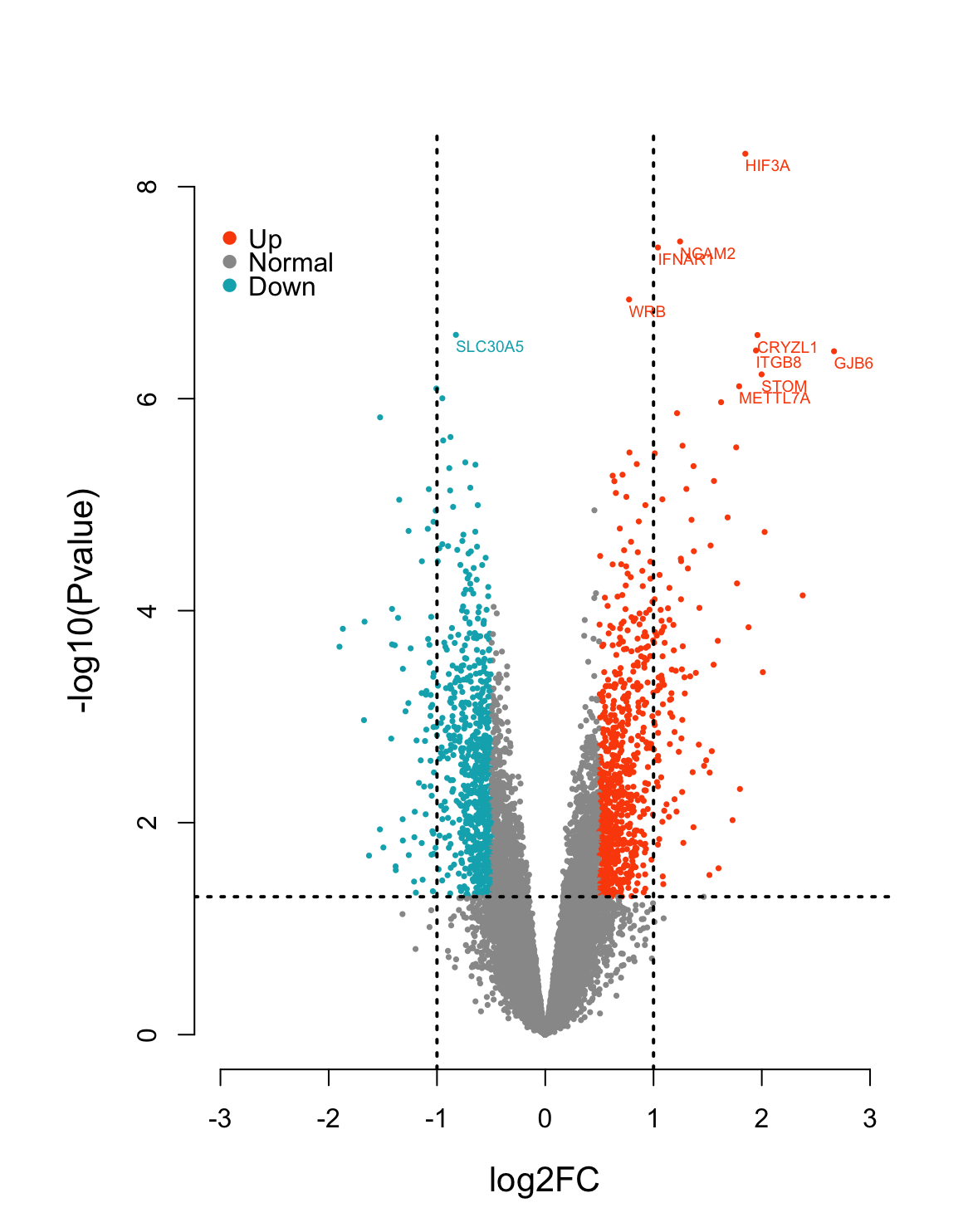

Supplement: Supplementary file 1 [file ijms-24-09980-s001.zip › Figure S2_a_CBC.tiff]

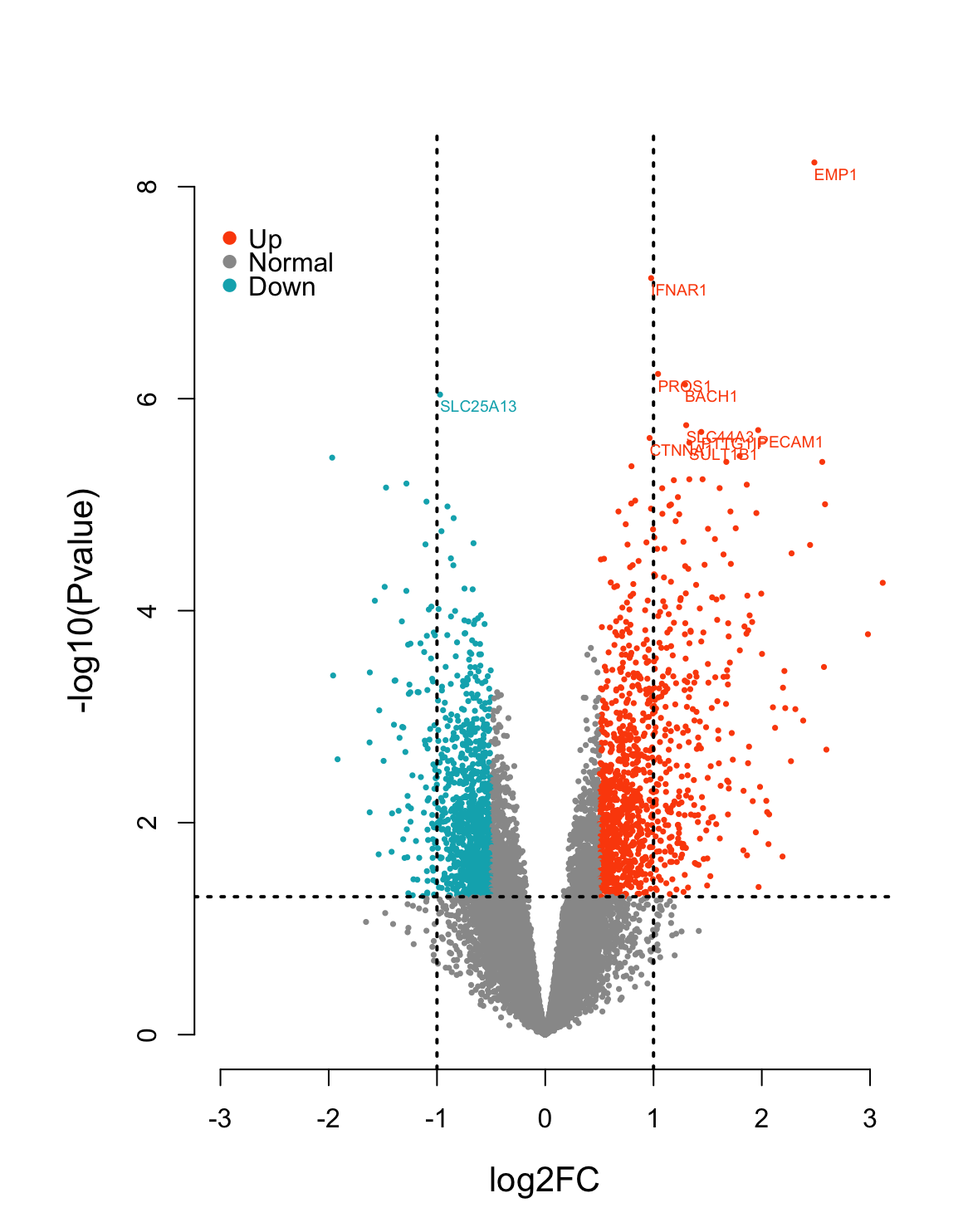

Supplement: Supplementary file 1 [file ijms-24-09980-s001.zip › Figure S2_b_DFC.tiff]

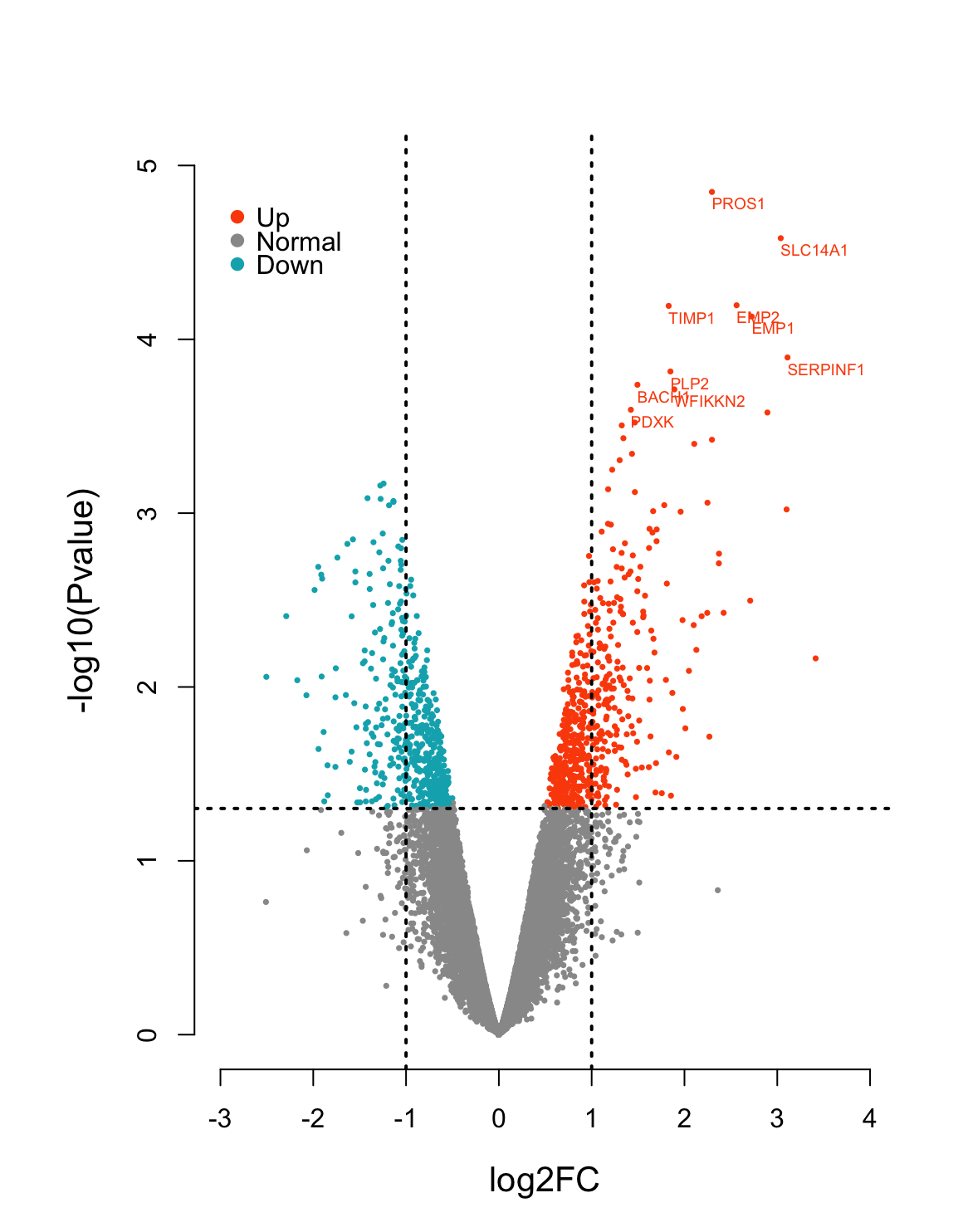

Supplement: Supplementary file 1 [file ijms-24-09980-s001.zip › Figure S2_c_HIP.tiff]

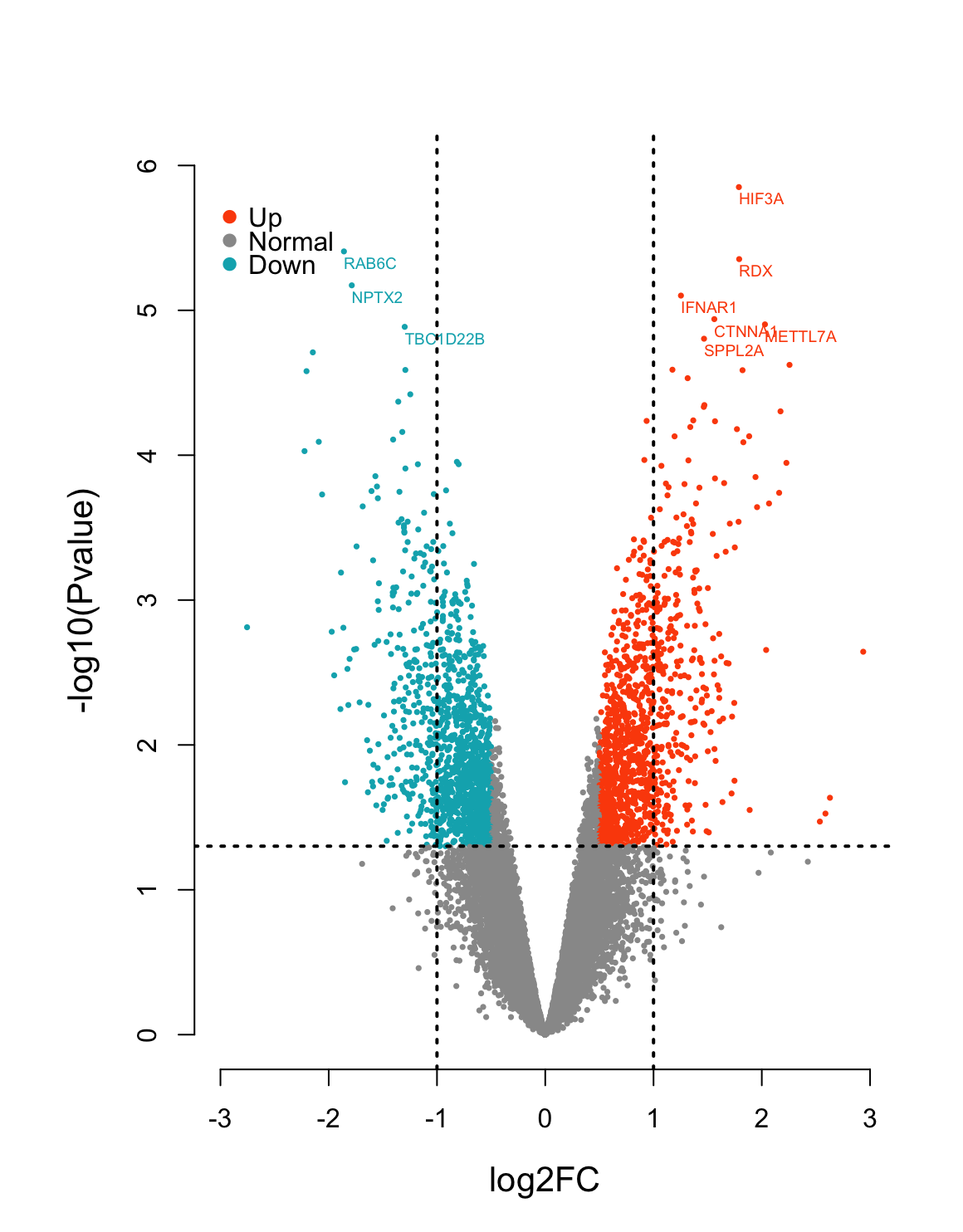

Supplement: Supplementary file 1 [file ijms-24-09980-s001.zip › Figure S2_d_ITC.tiff]

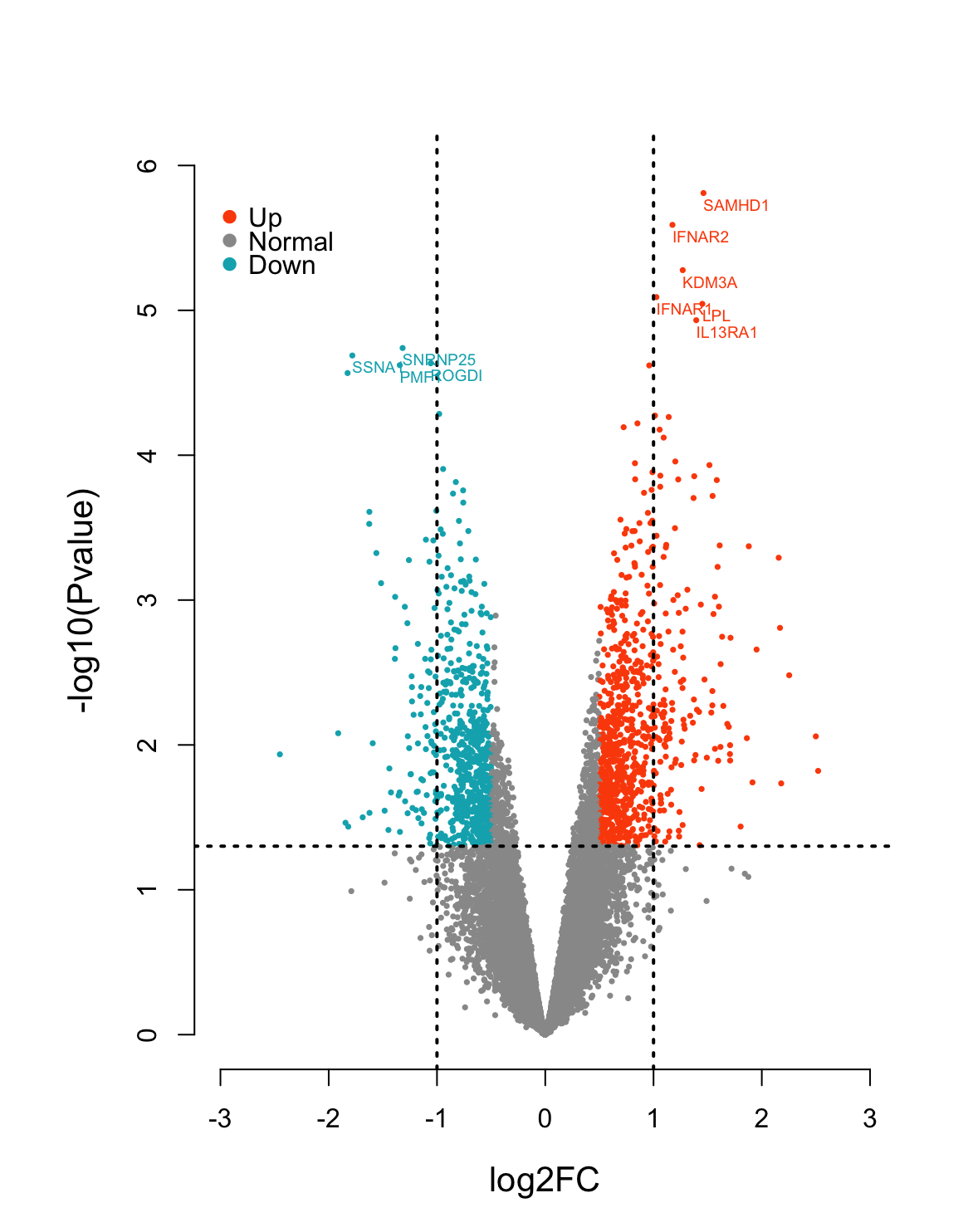

Supplement: Supplementary file 1 [file ijms-24-09980-s001.zip › Figure S2_e_OFC.tiff]

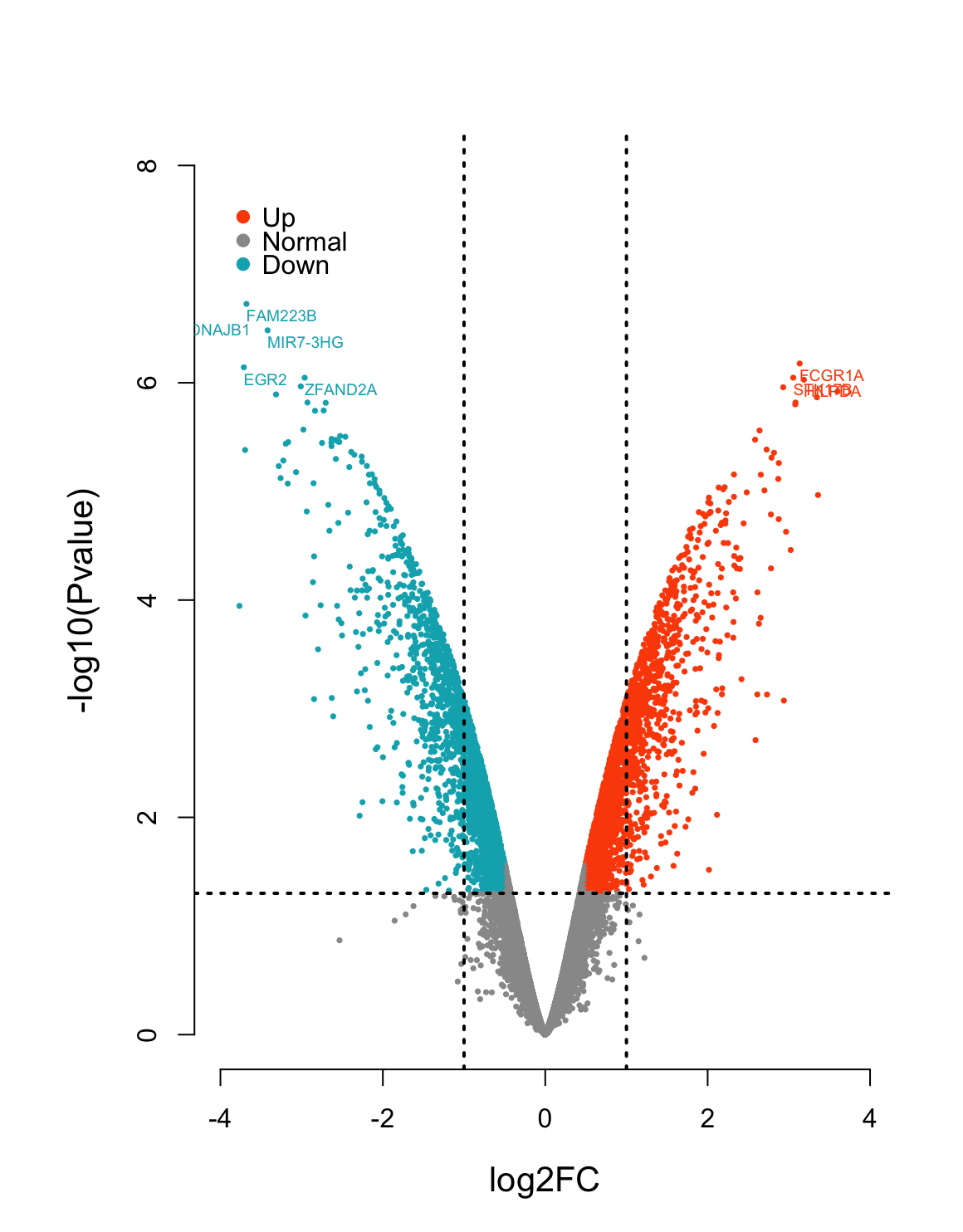

Supplement: Supplementary file 1 [file ijms-24-09980-s001.zip › Figure S2_f_S1C.tiff]

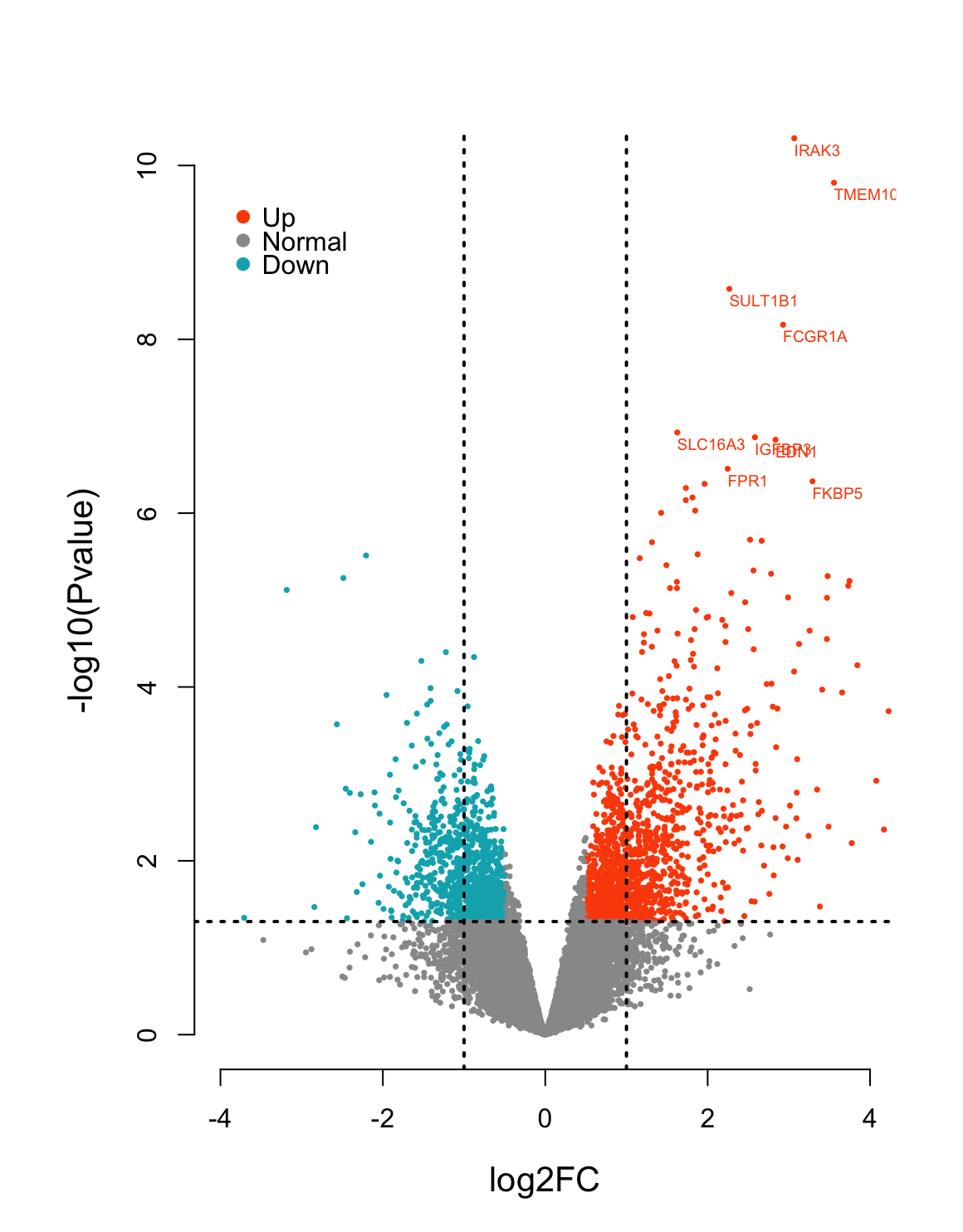

Supplement: Supplementary file 1 [file ijms-24-09980-s001.zip › Figure S2_g_V1C.tiff]

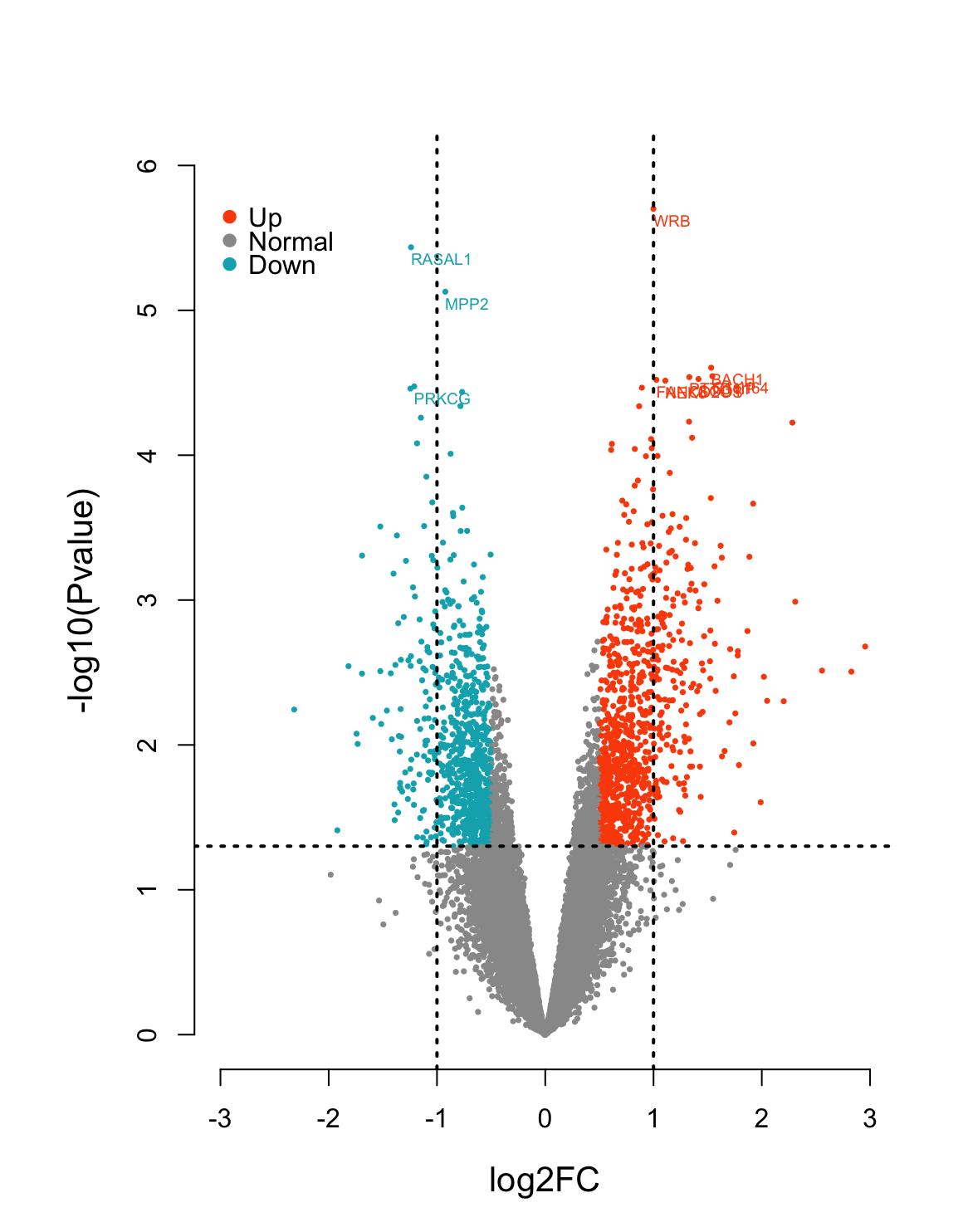

Supplement: Supplementary file 1 [file ijms-24-09980-s001.zip › Figure S2_h_VFC.tiff]

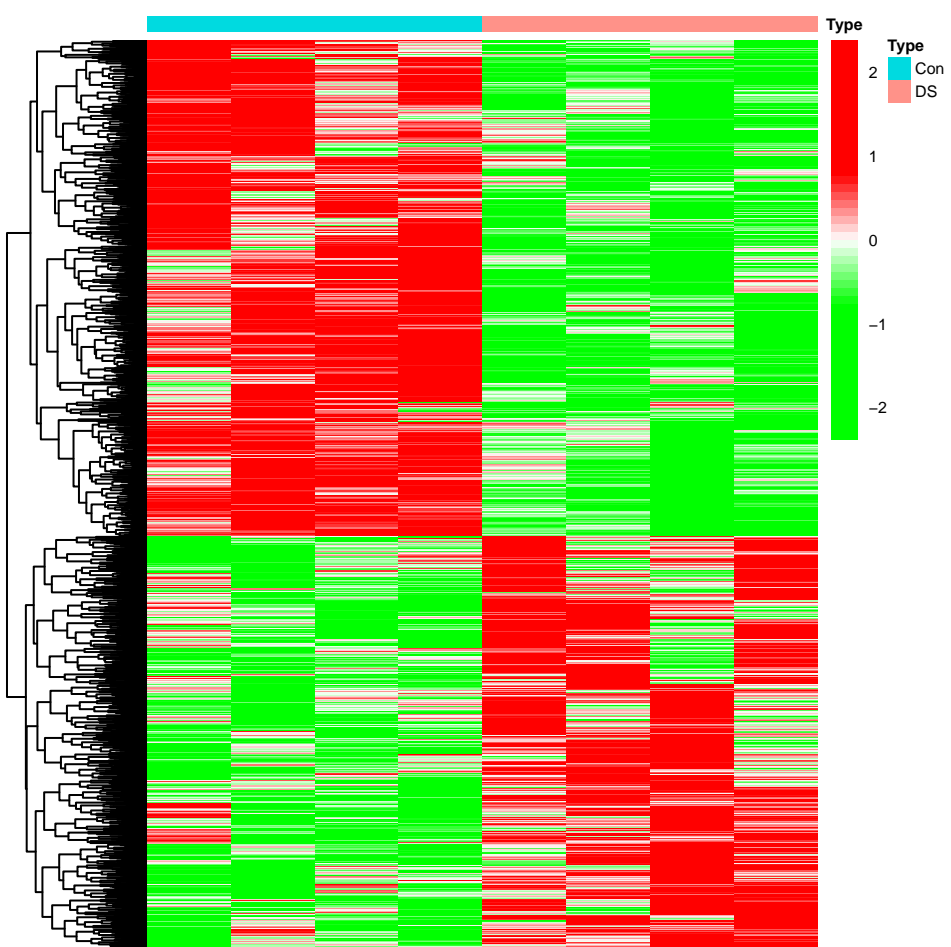

Supplement: Supplementary file 1 [file ijms-24-09980-s001.zip › Figure S3_a_Fetal.pdf]

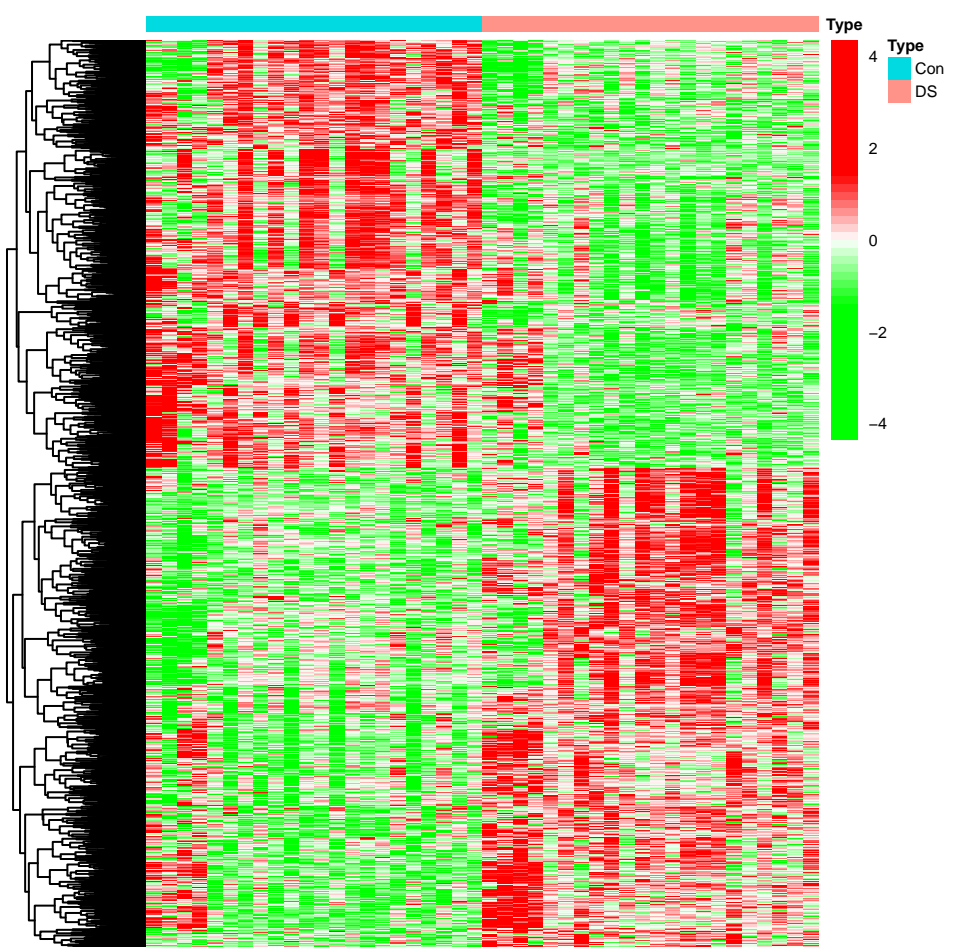

Supplement: Supplementary file 1 [file ijms-24-09980-s001.zip › Figure S3_b_Infant.pdf]

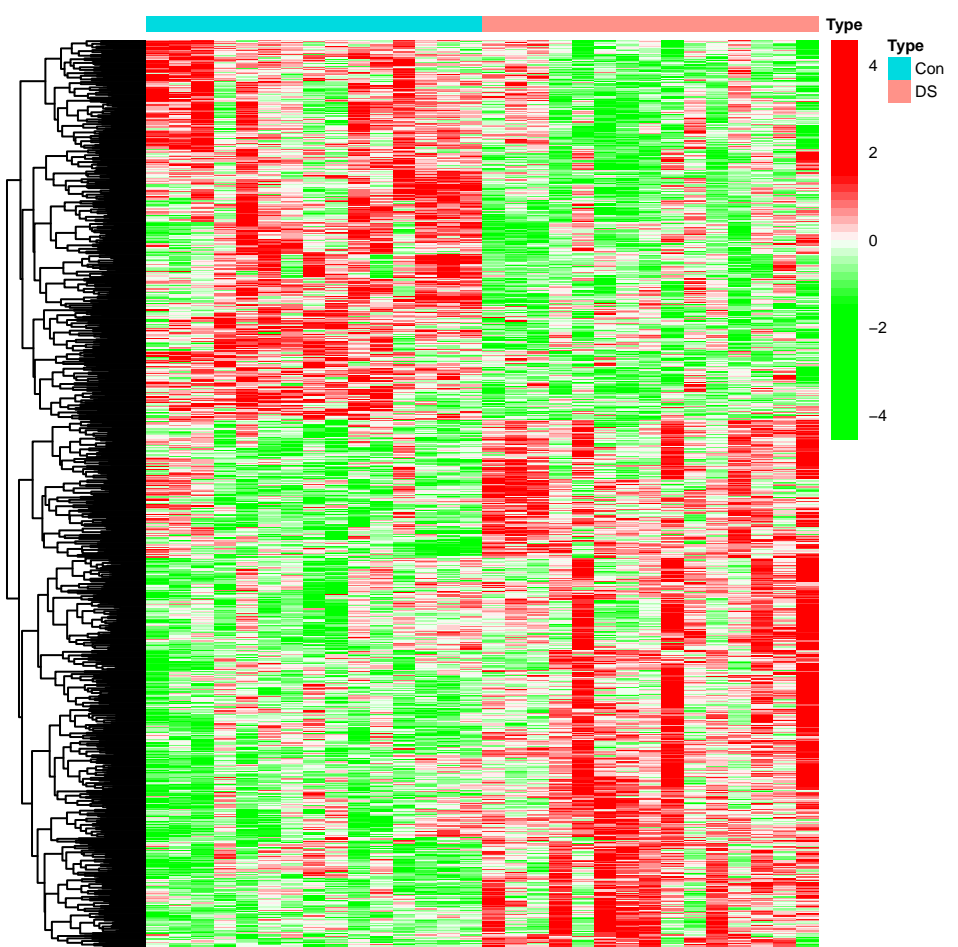

Supplement: Supplementary file 1 [file ijms-24-09980-s001.zip › Figure S3_c_Child.pdf]

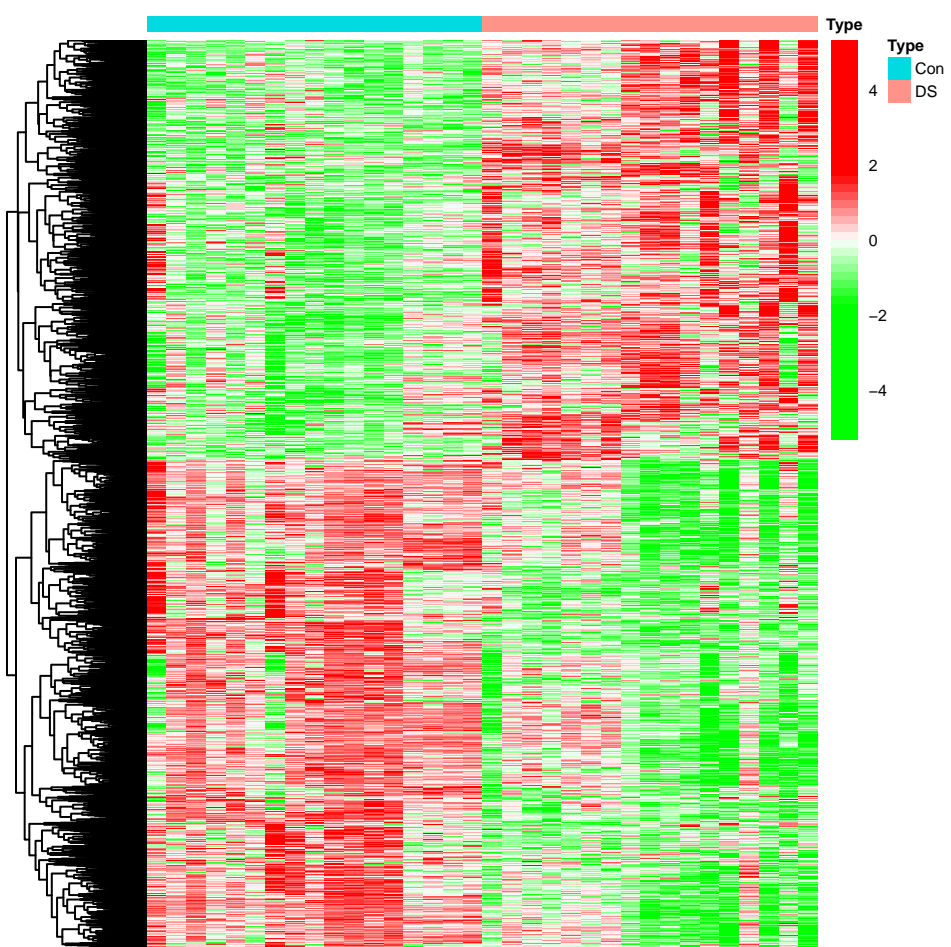

Supplement: Supplementary file 1 [file ijms-24-09980-s001.zip › Figure S3_d_Adult.pdf]

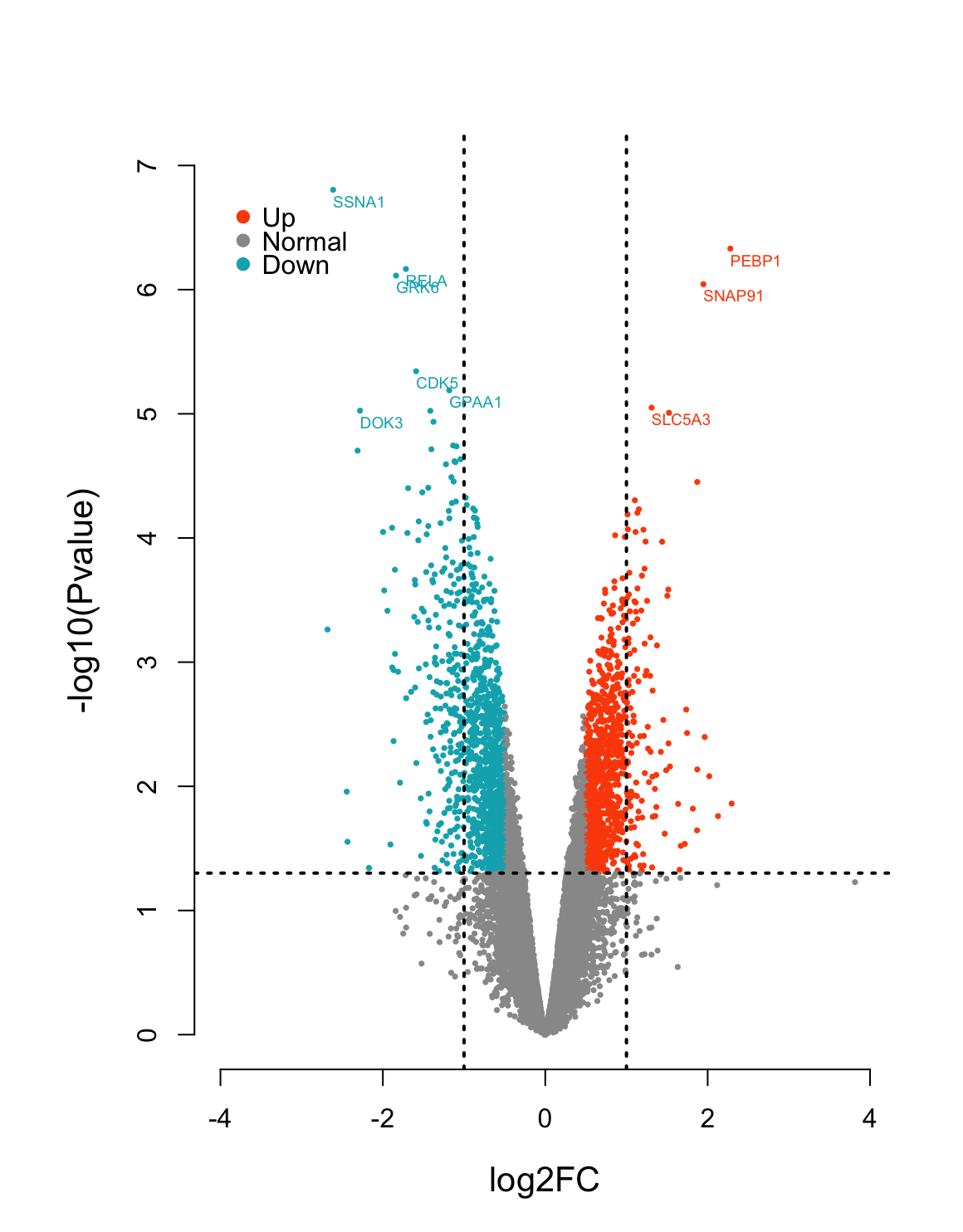

Supplement: Supplementary file 1 [file ijms-24-09980-s001.zip › figure S4_a_Fetal.tiff]

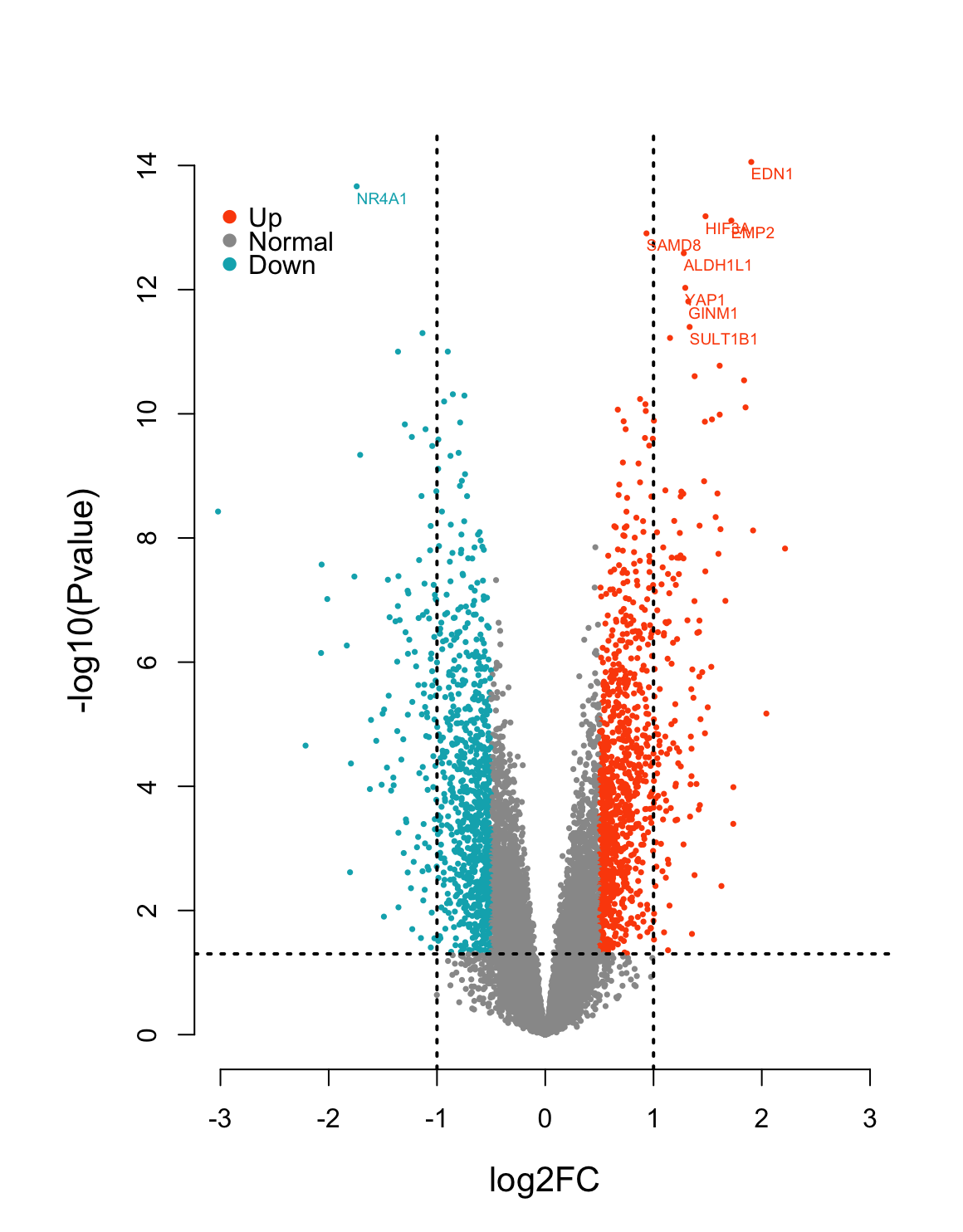

Supplement: Supplementary file 1 [file ijms-24-09980-s001.zip › Figure S4_b_Infant.tiff]

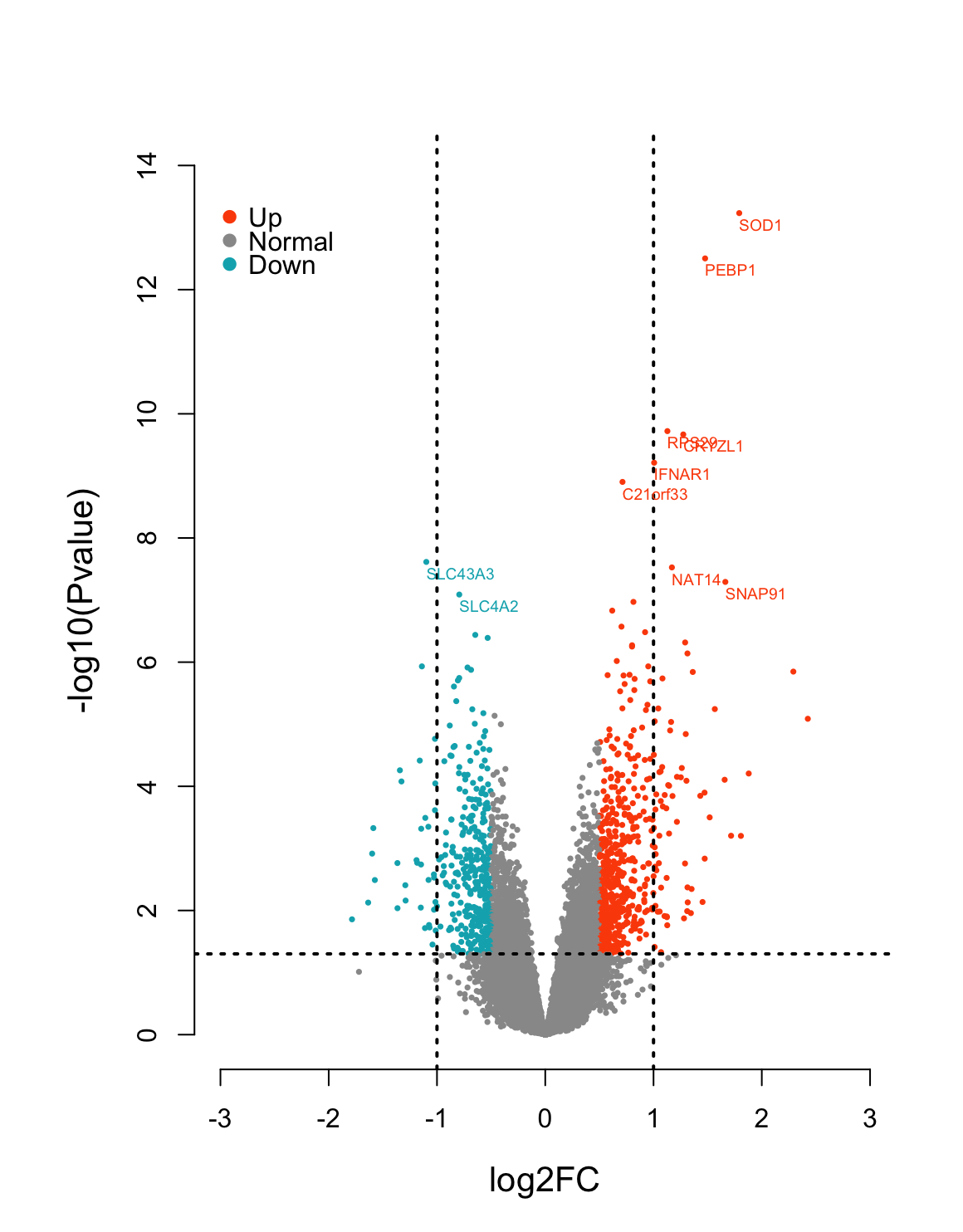

Supplement: Supplementary file 1 [file ijms-24-09980-s001.zip › Figure S4_c_Child.tiff]

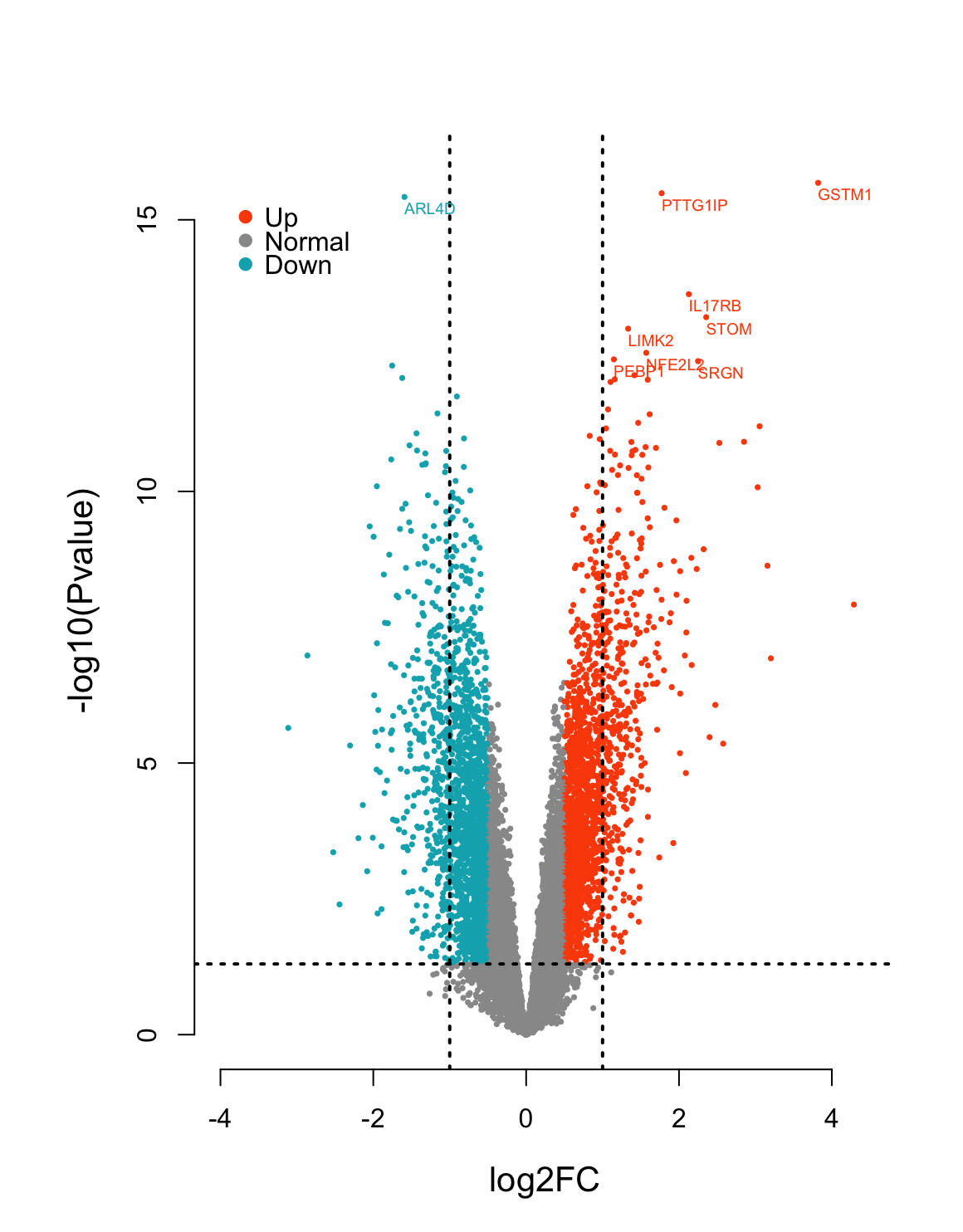

Supplement: Supplementary file 1 [file ijms-24-09980-s001.zip › Figure S4_d_Adult.tiff]

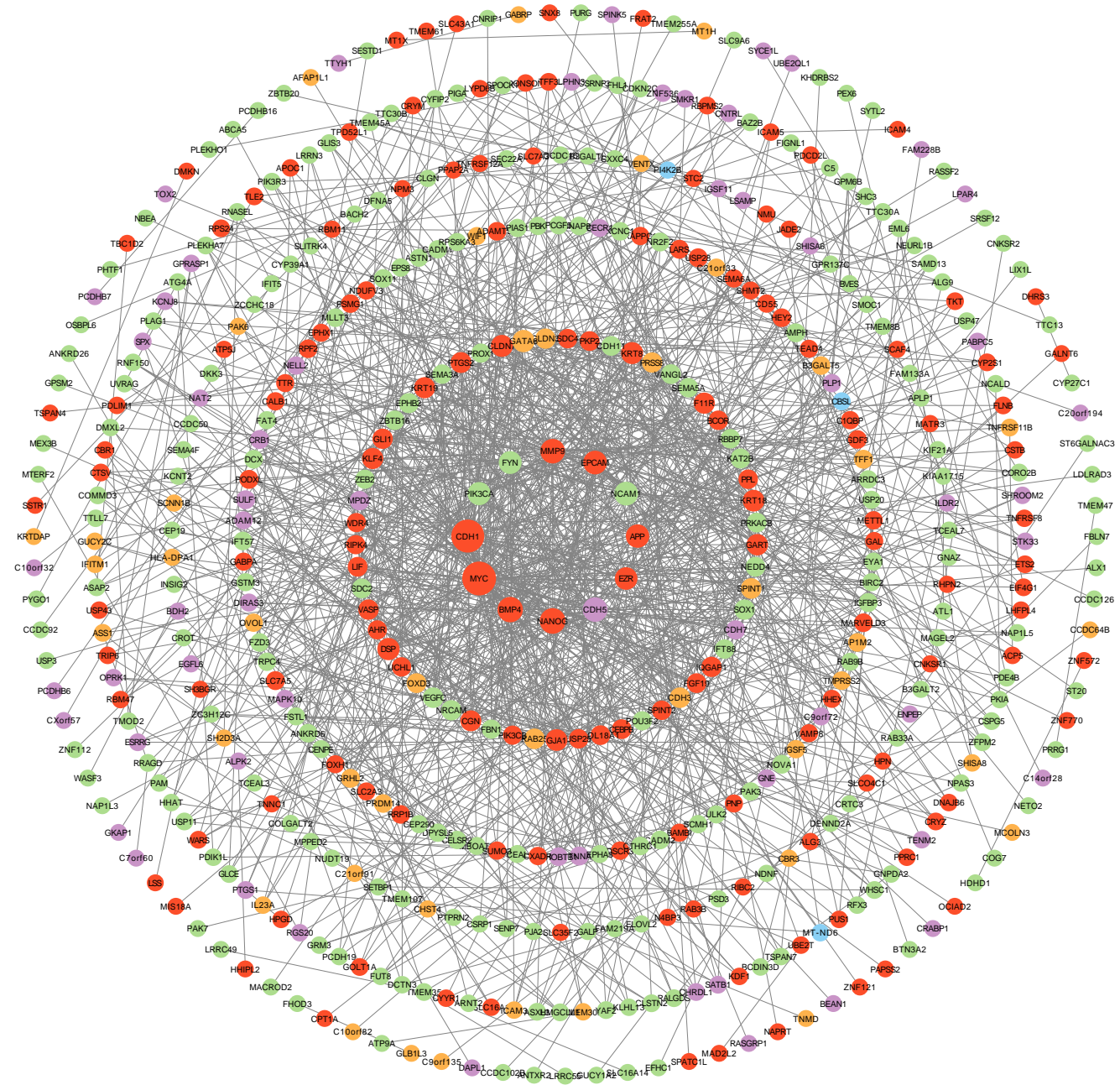

Supplement: Supplementary file 1 [file ijms-24-09980-s001.zip › Figure S5.pdf]
